# Supplementary material for: Early childhood risk and protective factors and their association with adolescent sexual behaviors: A Latent Class Analysis
Source: PLoS One. 2025 Oct 6;20(10):e0332247. doi: 10.1371/journal.pone.0332247 (PMC12500099; doi:10.1371/journal.pone.0332247)
Supplement: S1 Table — (DOCX) [file pone.0332247.s001.docx]

**Supporting Information: S1 Table**

**S1 Table**

*Descriptive Statistics for Original Continuous Variables*

| Variable | *N* | Sample Mean (*SD)* | Range |
| --- | --- | --- | --- |
| ***Early Childhood Risk and Protective Factors*** |  |  |  |
| Children Self-regulation (Y5) | 2,435 | 41.04 (6.26) | 15 – 50 |
| Maternal Warmth (Y5) | 1,814 | 6.17 (2.05) | 0 – 8 |
| Neighborhood Efficacy (Y5) | 2,745 | 21.38 (6.25) | 0 – 30 |
| Mother Impulsivity (Y5) | 3,195 | 4.65 (1.3) | 0 – 6 |
| Father Impulsivity (Y5) | 2,435 | 4.43 (1.46) | 0 – 6 |

*Note*. Means and standard deviations are reported for continuous variables. Y5 = Year 5.
