# Supplementary material for: Early childhood risk and protective factors and their association with adolescent sexual behaviors: A Latent Class Analysis
Source: PLoS One. 2025 Oct 6;20(10):e0332247. doi: 10.1371/journal.pone.0332247 (PMC12500099; doi:10.1371/journal.pone.0332247)
Supplement: S2 Table — (DOCX) [file pone.0332247.s002.docx]

**Supporting Information: S2 Table**

**S2 Table**

*Preliminary Analyses of Latent Profile Analysis with Continuous Variables (4-profile model)*

| **Latent Profiles** | **Indicator Variables** | | | | | | | | |
| --- | --- | --- | --- | --- | --- | --- | --- | --- | --- |
|  | Self-regulation | Parental Warmth | Neighborhood Cohesion | Education (M) | Poverty Status (M) | Education (F) | Poverty Status (F) | Impulsivity (M) | Impulsivity (F) |
| **1-class** | 10.77 | 5.73 | 29.50 | 2.08 | 2.55 | 2.11 | 4.99 | 6.53 | 6.01 |
| **2-class** | 10.20 | 5.76 | 30.16 | 1.95 | 1.97 | 1.76 | 1.47 | 6.43 | 6.03 |
| **3-class** | 8.94 | 6.09 | 30.93 | 2.21 | 2.63 | 2.07 | 2.99 | 6.58 | 6.40 |
| **4-class** | 8.29 | 6.33 | 31.58 | 2.38 | 3.01 | 2.30 | 4.00 | 6.63 | 6.62 |

*Note.* The table reports the mean of the indicator within each latent profile. M = Mother; F = Father.
