# Supplementary material for: Early childhood risk and protective factors and their association with adolescent sexual behaviors: A Latent Class Analysis
Source: PLoS One. 2025 Oct 6;20(10):e0332247. doi: 10.1371/journal.pone.0332247 (PMC12500099; doi:10.1371/journal.pone.0332247)
Supplement: S3 Table — (DOCX) [file pone.0332247.s003.docx]

**Supporting Information: S3 Table**

**S3 Table**

*Latent Class Prevalence and Item-Response Probabilities for Four-Class Model of Protective and Risky Indicators*

| Indicators | **Class 1:** Multidimensional Protective | **Class 2:** Socioeconomic Risk | **Class 3:** Neighborhood Risk | | **Class 4:**  Low Father Education |  |
| --- | --- | --- | --- | --- | --- | --- |
|  | **Latent Class Prevalences** | | | | |  |
|  | 21.5% | 59.2% | 7.3% | 12% | | |
|  | **Item-Response Probabilities** | | | | |  |
| **Children’s Self-regulation** |  |  |  | |  |  |
| Low | .29 | .51 | **.62** | | .35 |  |
| High | **.71** | .49 | .38 | | **.65** |  |
| **Maternal Warmth** |  |  |  | |  |  |
| Low | .30 | .52 | .53 | | .32 |  |
| High | **.70** | .48 | .47 | | **.68** |  |
| **Neighborhood Collective Efficacy** |  |  |  | |  |  |
| Low | .18 | .51 | 1 | | .12 |  |
| High | **.82** | .49 | 0 | | **.88** |  |
| **Mother Education** |  |  |  | |  |  |
| High school or below | .09 | **.70** | .37 | | .44 |  |
| College or higher | **.91** | .30 | **.63** | | .56 |  |
| **Mother Poverty Status** |  |  |  | |  |  |
| Poor | .19 | **.98** | .55 | | .33 |  |
| Non-poor | **.81** | .02 | .45 | | **.67** |  |
| **Father Education** |  |  |  | |  |  |
| High school or below | 0 | **.77** | .46 | | **.67** |  |
| College or higher | **1** | .23 | .54 | | .33 |  |
| **Father Poverty Status** |  |  |  | |  |  |
| Poor | .05 | **.80** | .23 | | .28 |  |
| Non-poor | **.95** | .20 | **.77** | | **.72** |  |
| **Mother Impulsivity** |  |  |  | |  |  |
| High | .32 | .57 | **.70** | | .38 |  |
| Low | **.68** | .43 | .30 | | **.62** |  |
| **Father Impulsivity** |  |  |  | |  |  |
| High | .33 | **.65** | .53 | | .53 |  |
| Low | **.67** | .35 | .47 | | .47 |  |

*Note*. Item-response probabilities greater than .60 are in bold to facilitate interpretation.
