# Supplementary material for: Early childhood risk and protective factors and their association with adolescent sexual behaviors: A Latent Class Analysis
Source: PLoS One. 2025 Oct 6;20(10):e0332247. doi: 10.1371/journal.pone.0332247 (PMC12500099; doi:10.1371/journal.pone.0332247)
Supplement: S4 Table — (DOCX) [file pone.0332247.s004.docx]

**Supporting Information: S4 Table**

**S4 Table**

*Average Latent Class Probabilities for the Four-Class Model of Protective and Risky Indicators*

| Assigned Classes | Class 1 | Class 2 | Class 3 | Class 4 |
| --- | --- | --- | --- | --- |
| Class 1 | **0.76** | 0.02 | 0.05 | 0.17 |
| Class 2 | 0.02 | **0.83** | 0.07 | 0.08 |
| Class 3 | 0.12 | 0.13 | **0.63** | 0.13 |
| Class 4 | 0.08 | 0.10 | 0.04 | **0.78** |

*Note*. Values represent average posterior probabilities for participants assigned to each latent class (rows) of actually belonging to each class (columns). Diagonal values indicate average classification certainty.
