# Supplementary material for: Early childhood risk and protective factors and their association with adolescent sexual behaviors: A Latent Class Analysis
Source: PLoS One. 2025 Oct 6;20(10):e0332247. doi: 10.1371/journal.pone.0332247 (PMC12500099; doi:10.1371/journal.pone.0332247)
Supplement: S1 File — (DOCX) [file pone.0332247.s005.docx]

**S1 File:**

**Mplus and R Coding**

This file contains the annotated Mplus code used to conduct the Latent Class Analysis (LCA) and the BCH method for examining distal outcomes. The syntax is organized into three parts:

**Part 1: Latent Class Analysis: Class Enumeration for 4-Class Solution**

TITLE: LCA 4-Class Class Enumeration;

DATA:

File = FFCW_FilterCases_NOLabels.csv;

VARIABLE:

NAMES =

idnum csex cage5 cage15 mage_5 fage_5 mrace mari

likegirl likeboy eversexC eversexA usecon mulsex firstsex

sr1 – sr25 pw1 – pw8 neibor1 – neibor10

mses fses medu fedu mip_1 mip_2 fip_1 fip_2

eversex SameSexA sr_sum pw_sum nei_sum mip fip

sr_sum_D pw_sum_D neisum_D mip_D fip_D

Zsrsum Zpwsum Zneisum Zmipsum Zfipsum;

MISSING ARE ALL (-999);

USEVARIABLES =

sr_sum_D pw_sum_D neisum_D medu mses fedu fses mip_D fip_D;

CLASSES = C(4);

CATEGORICAL =

sr_sum_D pw_sum_D neisum_D medu mses fedu fses mip_D fip_D;

ANALYSIS:

TYPE = MIXTURE;

STARTS = 500 100;

!PROCESSORS = 4;

!COVERAGE = 0;

MODEL:

%OVERALL%

[sr_sum_D$1 pw_sum_D$1 neisum_D$1 medu$1 mses$1 fedu$1 fses$1 mip_D$1 fip_D$1];

%C#1%

[sr_sum_D$1 pw_sum_D$1 neisum_D$1 medu$1 mses$1 fedu$1 fses$1 mip_D$1 fip_D$1];

%C#2%

[sr_sum_D$1 pw_sum_D$1 neisum_D$1 medu$1 mses$1 fedu$1 fses$1 mip_D$1 fip_D$1];

%C#3%

[sr_sum_D$1 pw_sum_D$1 neisum_D$1 medu$1 mses$1 fedu$1 fses$1 mip_D$1 fip_D$1];

%C#4%

[sr_sum_D$1 pw_sum_D$1 neisum_D$1 medu$1 mses$1 fedu$1 fses$1 mip_D$1 fip_D$1];

OUTPUT:

TECH10 TECH11 TECH14;

**Part 2: BCH Approach: Save Class Probability**

TITLE: LCA 4-Class Class Probability;

DATA:

File = FFCW_FilterCases_NOLabels.csv;

VARIABLE:

NAMES =

idnum csex cage5 cage15 mage_5 fage_5 mrace mari

likegirl likeboy eversexC eversexA usecon mulsex firstsex

sr1 – sr25 pw1 – pw8 neibor1 – neibor10

mses fses medu fedu mip_1 mip_2 fip_1 fip_2

eversex SameSexA sr_sum pw_sum nei_sum mip fip

sr_sum_D pw_sum_D neisum_D mip_D fip_D

Zsrsum Zpwsum Zneisum Zmipsum Zfipsum;

MISSING ARE ALL (-999);

USEVARIABLES =

sr_sum_D pw_sum_D neisum_D medu mses fedu fses mip_D fip_D;

CLASSES = C(4);

CATEGORICAL =

sr_sum_D pw_sum_D neisum_D medu mses fedu fses mip_D fip_D;

AUXILIARY =

csex cage5 mage_5 fage_5 mari mrace SameSexA

eversex firstsex usecon mulsex;

ANALYSIS:

TYPE = MIXTURE;

STARTS = 500 100;

!PROCESSORS = 4;

!COVERAGE = 0;

MODEL:

%OVERALL%

[sr_sum_D$1 pw_sum_D$1 neisum_D$1 medu$1 mses$1 fedu$1 fses$1 mip_D$1 fip_D$1];

%C#1%

[sr_sum_D$1 pw_sum_D$1 neisum_D$1 medu$1 mses$1 fedu$1 fses$1 mip_D$1 fip_D$1];

%C#2%

[sr_sum_D$1 pw_sum_D$1 neisum_D$1 medu$1 mses$1 fedu$1 fses$1 mip_D$1 fip_D$1];

%C#3%

[sr_sum_D$1 pw_sum_D$1 neisum_D$1 medu$1 mses$1 fedu$1 fses$1 mip_D$1 fip_D$1];

%C#4%

[sr_sum_D$1 pw_sum_D$1 neisum_D$1 medu$1 mses$1 fedu$1 fses$1 mip_D$1 fip_D$1];

OUTPUT:

TECH10 TECH11 TECH14;

SAVEDATA:

FILE IS 4CLASS.dat;

SAVE = CPROB;

MISSFLAG = 999;

FORMAT = FREE;

**Part 3: BCH Approach: Included predictors (covariates) and distal outcomes**

TITLE: LCA 4-Class BCH Outcomes;

DATA:

File is 4CLASS.dat;

DEFINE:

if MRACE == 1 then White = 1;

if MRACE == 2 then White = 0;

if MRACE == 3 then White = 0;

if MRACE == 4 then White = 0;

if MRACE == 1 then Hispanic = 0;

if MRACE == 2 then Hispanic = 0;

if MRACE == 3 then Hispanic = 1;

if MRACE == 4 then Hispanic = 0;

if MRACE == 1 then Others = 0;

if MRACE == 2 then Others = 0;

if MRACE == 3 then Others = 0;

if MRACE == 4 then Others = 1;

!Black is the reference group

VARIABLE:

NAMES =

SR_SUM_D PW_SUM_D NEISUM_D

MEDU MSES FEDU FSES MIP_D FIP_D

CSEX CAGE5 MAGE_5 FAGE_5 MARI MRACE SAMESEXA

EVERSEX FIRSTSEX USECON MULSEX

CPROB1 CPROB2 CPROB3 CPROB4 CL;

MISSING ARE ALL (999);

USEVARIABLES =

!demographic correlates

CSEX CAGE5 MAGE_5 FAGE_5 MARI SAMESEXA

!outcomes

EVERSEX FIRSTSEX USECON MULSEX CL

! define race

White Hispanic Others;

CLASSES = C(4);

nominal = CL;

AUXILIARY =

EVERSEX (BCH)

FIRSTSEX (BCH)

USECON (BCH)

MULSEX (BCH)

;

ANALYSIS:

TYPE = MIXTURE;

estimator = MLR;

STARTS = 0;

INTEGRATION = MONTECARLO;

ALGORITHM=INTEGRATION;

MODEL:

%OVERALL%

C ON CSEX CAGE5 MAGE_5 FAGE_5 MARI SAMESEXA White Hispanic Others;

CSEX;

CAGE5;

MARI;

MAGE_5;

FAGE_5;

SAMESEXA;

White;

Hispanic;

Others;

!logi in the crops in row

%c#1%

[CL#1@2.837];

[CL#2@0.376];

[CL#3@-0.088];

%c#2%

[CL#1@-0.964];

[CL#2@3.698];

[CL#3@-0.286];

%c#3%

[CL#1@0.698];

[CL#2@2.051];

[CL#3@2.178];

%c#4%

[CL#1@-0.943];

[CL#2@-0.664];

[CL#3@-2.296];

OUTPUT:

standardized;
