# Supplementary material for: Early childhood risk and protective factors and their association with adolescent sexual behaviors: A Latent Class Analysis
Source: PLoS One. 2025 Oct 6;20(10):e0332247. doi: 10.1371/journal.pone.0332247 (PMC12500099; doi:10.1371/journal.pone.0332247)
Supplement: Attachment — Submitted filename: Comments for 04608.docx [file pone.0332247.s006.docx]

**Reviewer Comments on Manuscript PONE-D-25-04608**

Title: Early childhood risk and protective factors and their association with adolescent sexual behaviors: A Latent Class Analysis

**Recommendation: Minor Revision**

**General Assessment**

This manuscript offers a significant contribution to the literature on adolescent sexual behavior by examining how early childhood risk and protective factors, across ecological domains, shape sexual outcomes in adolescence. The use of a person-centered approach via Latent Class Analysis (LCA), in a diverse, longitudinal dataset, is both timely and methodologically appropriate. The theoretical framing—drawing from life history theory, the social-ecological model, and risk and resilience perspectives—adds conceptual richness to the study.

The manuscript is well-written, logically structured, and thorough. However, certain areas require clarification or expansion, particularly around methodological choices and the interpretation of findings. With minor revisions, this work will be suitable for publication.

**Major Comments**

**Justification for Dichotomization of Indicators**

While the decision to dichotomize continuous indicators is acknowledged and justified by prior literature, it inherently reduces variability and interpretability. I recommend elaborating on this trade-off in the limitations or methods section, and possibly including supplementary information (e.g., original distributions) to reassure readers of the robustness of class distinctions.

**Interpretation of Smaller Classes**

The discussion primarily focuses on the SES Risk and Multidimensional Protective classes. However, the Neighborhood Risk and Low Father Education classes offer valuable insights. A more in-depth interpretation of these profiles and their implications for targeted interventions would strengthen the discussion.

**Policy and Practice Implications**

The conclusion briefly mentions the need for early interventions. To enhance its impact, I suggest identifying specific types of interventions (e.g., neighborhood-level programs, parenting education, school-based support for children from low SES backgrounds). This would make the findings more actionable for practitioners and policymakers.

**Model Selection Clarification**

Although the manuscript explains why the 4-class model was chosen over the 3-class alternative, readers less familiar with LCA might benefit from a more explicit explanation of why interpretability was prioritized over fit statistics (e.g., AIC/BIC).

**Minor Comments**

**Title & Abstract:** Clear and appropriate. However, the abstract conclusion could be more actionable—consider specifying the nature of "targeted interventions."

**Theoretical Framework:** Strong and well-integrated, but could be tightened for brevity, especially in overlapping explanations of life history theory.

**Figures & Tables:** Ensure that Figure 1 is clearly labeled and legible, especially for readers interpreting class probabilities.

**Limitations:** Consider mentioning unmeasured contextual variables (e.g., peer influences, media exposure) that may also shape adolescent sexual behavior.

**Conclusion**

This is a well-executed and theoretically sound manuscript that aligns with PLOS ONE's interdisciplinary scope. It has the potential to inform early preventive strategies in adolescent sexual health and development. I recommend minor revisions aimed at expanding interpretation, clarifying methodological decisions, and enhancing the practical relevance of the findings.
